# Previous Sternotomy as a Risk Factor in Minimally Invasive Mitral Valve Surgery

**DOI:** 10.3389/fsurg.2018.00005

**Published:** 2018-02-09

**Authors:** Jan-Philipp Minol, Payam Akhyari, Udo Boeken, Alexander Albert, Philipp Rellecke, Vanessa Dimitrova, Stephan Urs Sixt, Hiroyuki Kamiya, Artur Lichtenberg

**Affiliations:** ^1^Department of Cardiovascular Surgery, Universitätsklinikum Düsseldorf, Düsseldorf, Germany; ^2^Department of Anaesthesiology, Universitätsklinikum Düsseldorf, Düsseldorf, Germany

**Keywords:** cardiac surgery, minimally invasive, lateral thoracotomy, mitral valve, redo surgery

## Abstract

**Background:**

Cardiac redo surgery, especially after a full sternotomy, is considered a high-risk procedure. Minimally invasive mitral valve surgery (MIMVS) is a potential therapeutic approach. However, current developments in interventional cardiology necessitate additional discussion regarding the therapy of choice in high-risk patients. In this context, it is necessary to clarify the perioperative and postoperative risks induced by the factor *previous sternotomy* in the setting of MIMVS. Thus, we present a comparative study analyzing the outcome of MIMVS after previous sternotomy vs. primary operation.

**Methods:**

We identified 19 patients who received isolated or combined mitral valve (MV) surgery *via* the MIMVS approach after previous full sternotomy (PS group) and compared the results to those of a group of 357 patients who received primary MIMVS (non-PS group). After a propensity score analysis, groups of *n* = 15 and *n* = 131, respectively, were subjected to a comparative evaluation. A 1-year follow-up analysis of functional cardiac parameters and clinical symptoms was performed, accompanied by a Kaplan–Meier analysis.

**Results:**

Except for the rate of realized MV reconstructions (PS group: 53.8% vs. non-PS group: 85.5%; *p* = 0.011), no significant differences were to be noted within the intraoperative and early postoperative course. However, patients in the PS group experienced an increased intensive care unit stay length (PS group: 2 days, 95% CI, 1–8 vs. non-PS group: 1 day, 95% CI, 1–2; *p* = 0.072). The follow-up examinations revealed excellent functional and clinical outcomes for both groups. The Kaplan–Meier analysis displayed no significant difference regarding the postoperative mortality (*p* = 0.929) related to the patients at risk.

**Conclusion:**

A previous sternotomy remains a risk factor for MIMVS and demands special attention in the early postoperative period. Nevertheless, the early- and late-term results concerning the functional and clinical outcomes suggest that the MIMVS procedure is satisfactory, even after a full sternotomy.

## Introduction

Redo procedures after a previous sternotomy for cardiac surgery are considered challenging, with significant associated risk (1–9). A median resternotomy has a high rate of injury during the redo procedure, especially in patients with vascular structures that lie directly behind the sternum (ascending aorta, right ventricle, or patent coronary bypass grafts) (8–11).

Minimally invasive cardiac surgery *via* right lateral minithoracotomy [minimally invasive mitral valve surgery (MIMVS)] is a well-established procedure. Numerous studies have determined that it is at least equal to a full sternotomy with respect to surgical outcomes (1–4, 6). Even in high-risk patients, MIMVS demonstrated no inferiority compared to a sternotomy regarding the mortality, repair rate, and postoperative outcome, although MIMVS requires a longer cardiopulmonary bypass (CPB) time (12).

Nevertheless, currently, evolving strategies such as transcatheter mitral valve replacement (TMVR) have challenged the suitability and safety of operative procedures on the mitral valve (MV) *via* a right lateral minithoracotomy in the setting of a redo procedure. Current guidelines from the European Society of Cardiology and the European Association for Cardio-Thoracic Surgery suggest a percutaneous edge-to-edge procedure in patients with symptomatic severe primary and secondary mitral regurgitation, who are judged to be inoperable or at high surgical risk by a “heart team” and who have a life expectancy of greater than 1 year (recommendation class IIb, level of evidence C) (13).

In this context, some authors have identified inherent drawbacks of the MIMVS approach such as an increased rate of stroke and aortic dissections (14). This *MIMVS risk factor* affects the results of previous studies, which reported isolated groups of MIMVS after a previous sternotomy (7–9) or compared MIMVS and conventional MV surgery with a full sternotomy after a previous sternotomy (15–17).

As a result of the discussion regarding the indication of catheter-guided interventions in high-risk patients, we see the necessity to clarify the impact of the risk factor *previous sternotomy* on the outcome of MIMVS. Consequently, we report our experiences by comparatively analyzing two groups of patients with MIMVS with (PS) or without (non-PS) previous cardiac surgery *via* sternotomy after propensity score matching.

## Materials and Methods

Nineteen patients who had previously undergone cardiac surgery *via* full sternotomy (PS group) received cardiac redo surgery as an isolated MIMVS procedure or in combination with procedures on the tricuspid valve (TV) in our department as previously described in detail (18, 19). For preoperative planning, thoracic computed tomography (CT) was performed to assess the intrathoracic anatomy. Patients with extensive mitral annular calcifications received surgery *via* a repeated full sternotomy.

We sought to analyze the preoperative characteristics, the perioperative course, and the postoperative outcomes of this group compared with those of a group of 357 patients who received MIMVS as a primary operation during the same period in our department (non-PS group). Because of the inherent heterogeneity, both groups were subjected to propensity score matching that targeted the following parameters: sex, age, EF, and European System for Cardiac Outcome Risk Evaluation II (EuroSCORE II). The analysis design is described elsewhere (20). Because 21.1% of the PS group and 63.3% of the non-PS group did not meet the matching criteria, these patients were excluded from the final analysis. In the following comparative analysis and the representation of the follow-up analysis, we focus on the resulting groups of *n* = 15 (PS group) and *n* = 131 (non-PS group).

Throughout the article, categorical variables are expressed as proportions and were analyzed by Fisher’s exact test. Continuous variables are given as the mean ± SD and were subjected to Welch’s *t*-test after having passed the D’Agostino–Parson normality test. Non-normally distributed continuous variables are expressed as the median (25th–75th percentile) and were analyzed by the Mann–Whitney *U*-test.

All patients were contacted by telephone, interviewed to evaluate rehabilitation status and activity at 1 ear of follow-up, and invited for an onsite echocardiographic examination. Echocardiographic follow-up was performed by the attending cardiologist in selected cases where a visit to our center was not possible. The follow-up was subjected to a Kaplan–Meier analysis, supported by the Gehan–Breslow–Wilcoxon test. Differences were considered significant at *p* < 0.05. The analysis was performed using the InStat3 and Prism7 software programs (Graph Pad Software, La Jolla, CA, USA).

This study was approved by the local ethics committee (approval no.: 3650). The authors had full access to the data and take full responsibility for the integrity of the manuscript. All authors have read and agree to the manuscript as written.

## Results

All patients of the PS group received only one prior cardiothoracic procedure (isolated or combined), conducted *via* a full sternotomy. Coronary artery bypass had been performed in eight patients (53.9%). Six patients (40.0%) had received MV reconstruction; in two patients (13.3%), the MV had been replaced. Three patients (20.0%) displayed a history of aortic valve replacement. A TV reconstruction had been undertaken in one patient (6.7%). One patient (6.7%) underwent a repair of an atrial septal defect. The time interval between the initial operation and MIMVS was 50.1 ± 40.07 months.

A comparative analysis of the preoperative characteristics of the PS and non-PS group is presented in Table 1. Differences with respect to the EuroSCORE II values could not be fully equalized by propensity score matching (PS group: 4.56%, 95% CI, 3.09–8.16 vs. non-PS group: 1.86%, 95% CI, 1.02–3.35; *p* < 0.0001) at otherwise comparable preoperative comorbidities.

**Table 1:** Preoperative characteristics.

Preoperative characteristics	PS group	Non-PS group	*p*
*N*	15	131	
Female	3 (20.0%)	42 (32.0%)	1.000***
Body mass index ≥30 kg/m^2^	25.9 (2.8–0.7)	25.9 (4.7–0.4)	0.988*
NYHA	2.7 ± 0.8	2.4 ± 0.9	0.687**
Age (years)	68.0 (60.0–73.0)	66.0 (57.0–74.0)	0.955*
Endocarditis	1 (6.7%)	14 (10.7%)	1.000***
Atrial fibrillation	4 (26.7%)	47 (35.9%)	0.577***
Hypertension	12 (80.0%)	97 (74.0%)	0.761***
COPD	2 (13.3%)	19 (14.5%)	1.000***
Pulmonary hypertension	7 (46.7%)	61 (46.6%)	1.000***
IDDM	0	8 (6.1%)	1.000***
RF > II	3 (20.0%)	19 (14.5%)	0.701***
LVEF (%)	56 (50.0–61.5)	60 (52.0–64.0)	0.130*
MR > I	15 (100.0%)	122 (93.1%)	0.598***
MS > I	2 (13.3%)	9 (6.9%)	0.315***
Previous neurological events	3 (20.0%)	8 (6.1%)	0.088***
EuroSCORE II (%)	4.6 (3.1–8.2)	1.9 (1.1–3.4)	<0.0001*

*COPD, chronic obstructive pulmonary disease; EuroSCORE II, European System for Cardiac Outcome Risk Evaluation II; IDDM, insulin-dependent diabetes mellitus; LVEF, left ventricular ejection fraction; MR, mitral regurgitation; MS, mitral stenosis; NYHA, New York Heart Association index; RF, renal failure*.

*Mann–Whitney U-test.

**Welch’s t-test.

***Fisher’s exact test.

At admission, all 15 patients in the PS group exhibited MV regurgitation II or higher; four patients suffered from combined MV disease. During the described surgery at our department, MV repair was performed in seven patients (53.8%). All seven patients underwent annuloplasty; however, one patient also received P2 resection and three patients received neochordae insertion. Additional repair of the TV was performed in four cases (26.6%), *via* the De Vega technique in one and annuloplasty in three.

Intraoperative parameters (Table 2) displayed no significant difference regarding the duration of surgery (*p* = 0.206) or the duration of CPB (*p* = 0.106). The MV repair rate was significantly lower in the PS group (53.8 vs. 85.5% in the non-PS group; *p* < 0.011) after excluding two patients with previous MV replacement. No patient required conversion to full sternotomy. Eight patients (53.3%) in the PS group exhibited patent bypass grafts, and there was no injury to the existing grafts. In this subcohort, four patients (26.7%) with patent left internal thoracic artery grafts underwent surgery without cross-clamping, using ventricular fibrillation and moderate systemic hypothermia (28°C). This technique was also used in three patients without grafts but with intraoperative difficulties in clamping the aorta.

**Table 2:** Intraoperative course.

Intraoperative course	PS group	Non-PS group	*p*
*N*	15	131	
Combined MV/TV procedure	4 (26.6%)	32 (24.4%)	1.000***
Duration of surgery (min)	250 (210–323)	237 (199–270)	0.206*
Duration of CPB (min)	170 (136–215)	159 (132–185)	0.106*
Realized MV repairs	7 (53.8% of 13)	112 (85.5%)	0.011***

*MV, mitral valve; TV, tricuspid valve; CPB, cardiopulmonary bypass*.

*Mann–Whitney U-test.

***Fisher’s exact test.

During the early postoperative course, there were no significant differences regarding the analyzed parameters (Table 3). However, the most striking difference was the rate to the duration of the intensive care unit (ICU) stay (PS group: 2 days, 95% CI, 1–8 vs. non-PS group: 1 day, 95% CI, 1–2; *p* = 0.072).

**Table 3:** Early postoperative course.

Postoperative course	PS group	Non-PS group	*p*
*N*	15	131	
New-onset RF requiring dialysis	2 (13.3%)	6 (4.6%)	0.192***
Wound infection	0	8 (6.1%)	1.000***
Revision for bleeding	2 (13.3%)	7 (5.3%)	0.232***
Duration of ICU stay (days)	2.0 (1.0–8.0)	1.0 (1.0–2.0)	0.072*
Prolonged ventilation (>48 h)	1 (6.7%)	11 (8.4%)	1.000***
Postoperative neurological events	0	4 (3.1%)	1.000***

*ICU, intensive care unit; RF, renal failure*.

*Mann–Whitney U-test.

***Fisher’s exact test.

### Follow-up

**Figure 1:**
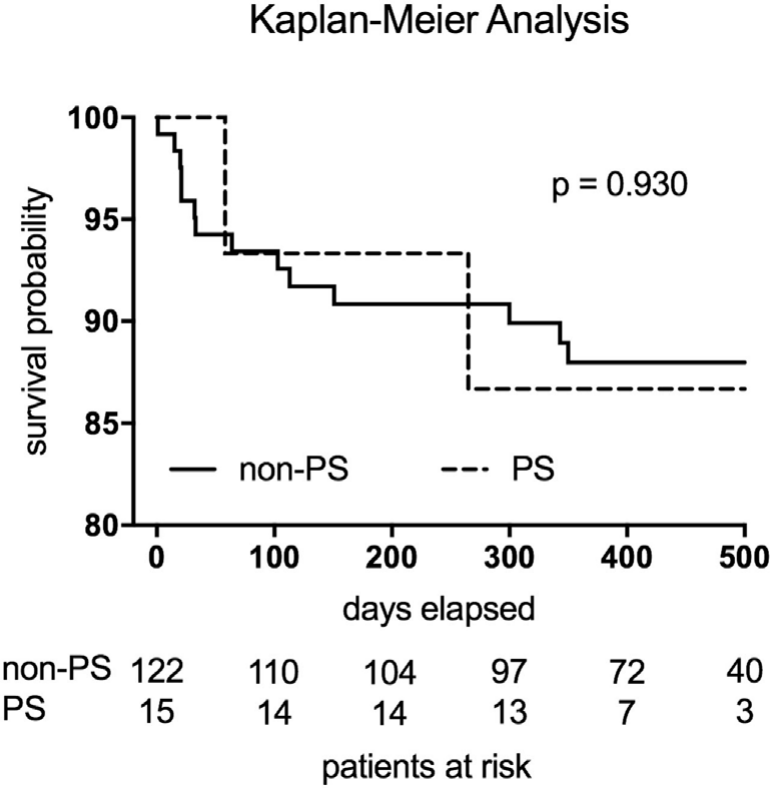
The Kaplan–Meier analysis revealed no significant difference regarding the postoperative mortality (*p* = 0.930) related to the at-risk patients. Two events (deaths) were noted within the PS group (broken line). The non-PS group displayed 14 events (continuous line).

Follow-up examinations regarding the PS and non-PS groups were completed in 100 vs. 92.3% (*p* = 0.597) of patients, respectively, and were conducted at 379 days (95% CI, 356–461; PS group) vs. 441 days (95% CI, 377–671; non-PS group) after surgery (*p* = 0.229; Table 4). In the non-PS group, 9 patients were lost to follow-up just after discharge and 14 patients died before follow-up examination. Clinical outcomes, as expressed by the New York Heart Association status and echocardiographic mitral function, were comparable between the groups. During follow-up, no cardiac-related reoperations were necessary within the PS group. The Kaplan–Meier analysis revealed no significant difference regarding the postoperative mortality (*p* = 0.930) related to the at-risk patients during the total follow-up period (Figure 1). In total, 2 events (deaths) were noted within the PS group vs. 14 events in the non-PS group. There was no significant difference between the 30-day mortality of the PS group and non-PS group (0 vs. 4.1%, respectively, *p* = 1.000) related to the at-risk patients.

**Table 4:** Follow-up examination.

Follow-up examination	PS group	Non-PS group	
*N*	13 (100%)	108 (92.3%)	0.597***
Δ*t* after surgery (days)	379 (356–461)	441 (377–671)	0.229*
MR > I°	1 (7.7%)	10 (9.3%)	1.000***
MR > II°	0	2 (1.9%)	1.000***
Cardiac-related re-operation	0	3 (2.8%)	1.000***
NYHA > II	1 (7.7%)	17 (15.7%)	0.689***

*MR, mitral regurgitation; NYHA, New York Heart Association index*.

*Mann–Whitney U-test.

***Fisher’s exact test.

## Discussion

Minimally invasive access *via* a right lateral minithoracotomy has been repeatedly reported as the technique of choice for cardiac redo surgery after previous sternotomy (5, 7–9, 12). Nevertheless, several studies have highlighted the risk of morbidity and mortality associated with cardiac redo surgery (1–9). In this context, patent coronary bypass grafts or dense adhesions significantly elevates the risk of relevant complications such as myocardial infarction and bleeding events. Right-sided minithoracotomy allows access adjacent to the area affected by previous surgery, thereby providing an advantage compared with repeated sternotomy.

We sought to report on our experience regarding this issue by comparing our MIMVS patients after a previous sternotomy with those without any previous cardiac surgery. As a key element of this approach, we conducted propensity score matching before the final analysis. Although this matching also included the factor *EuroSCORE II*, the values could not be fully equalized by propensity score matching. As the majority of the influencing factors were equalized, the differing EuroSCORE II has to be considered as a marker whether patients had undergone previous sternotomy.

In our cohort, MIMVS surgery after a previous sternotomy was associated with a CBP duration of 170 min (95% CI, 136–215 min). These results are comparable to those of the poststernotomy cohort of Murzi et al. (9) (160 ± 58 min), but differ from the somewhat shorter operative times reported by Seeburger et al. (8) (135 ± 41 min). In this context, it is important to note that 26.6% of our PS group required combined procedures in contrast to those from Murzi et al. (9) (19.1%) and Seeburger et al. (8) (11.1%). Moreover, a pre-existing aortic valve prosthesis negatively impacts subsequent MV surgery because of anatomic limitations on exposure. In our redo cohort, 20.0% of patients presented with previous AVR, compared with 18.7 and 0% in the cohorts of Murzi et al. (9) and Seeburger et al. (8), respectively. However, in our judgment, the more important fact is that despite this complexity, no significant difference was noted regarding the duration of CPB between the PS group and the non-PS group (170 min, 95% CI, 136–215 vs. 159 min, 95% CI, 132–185, respectively; *p* = 1.06). This result not only clearly supports the previously reported experience of an excellent access route to the MV without extensive cardiac mobilization as in redo procedures *via* sternotomy (7–9), but it also suggests that a redo surgery can be conducted within a comparable duration of CPB with the same surgical technique. The MIMVS inherently requires a prolonged CPB time compared with that in a conventional sternotomy (14). However, Moscarelli et al. stated in a meta-analysis that also in high-risk patients, MIMVS requires a longer CPB time compared to that in a standard sternotomy but results in similar mortality and even better postoperative outcomes (12).

One should also note the fact that seven patients (46.7%) in our PS group received surgery without cross-clamping using ventricular fibrillation and moderate systemic hypothermia (28°C). Romano et al. (21) reported on their experience with 450 patients undergoing redo MV surgery *via* right lateral minithoracotomy, with no patient receiving aortic cross-clamping. Romano et al. (21) used ventricular fibrillation at moderate hypothermia (core temperature 26°C) for 134 patients and beating heart surgery at mild hypothermia (core temperature 32°C) for the remaining 316 patients. The authors concluded that redo MIMVS on the beating heart is associated with shorter bypass time, reduced transfusion requirements, shorter postoperative ventilation, and a lower mortality rate. Botta et al. (10) supported this notion, suggesting that the beating heart approach avoids hypothermia and limits the subendocardial perfusion mismatch associated with ventricular fibrillation.

After excluding two patients with previous MV replacement, we observed a significantly lower MV repair rate in the PS group compared with that in the non-PS group (53.8 vs. 85.5%, respectively; *p* = 0.011). Murzi et al. (9) and Seeburger et al. (8) described repair rates of 30.6 and 67.7%, respectively. The differences observed here reflect the different proportions of patients undergoing a redo operation after previous MV repair. In our PS group, six patients (40%) presented with previous MV repair, while in the cohort reported by Seeburger et al. (8), only 17% of patients presented with previous MV repair. In contrast, in the cohort reported by Murzi et al. (9), 70.1% had a previous MV procedure. The negative impact of a failed previous MV repair during a surgical redo repair has already been described by Conradi et al. (22). Furthermore, in the setting of a redo operation, the MIMVS approach is associated with a significant increase in repair rate, compared with a sternotomy redo (42.0 vs. 8.8%, respectively; *p* = 0.003) (23). Taking all these aspects into consideration, a preceding MV procedure appears to be a more appropriate predictor of MV replacement than simply the choice of access route, with or without a previous sternotomy.

The rate of postoperative neurologic complications is a major issue when considering MIMVS generally and especially as a redo approach (14, 24–27). Svensson et al. revealed retrograde arterial cannulation, ventricular fibrillatory arrest, and incomplete deairing as risk factors in this scenario (24). Later, Murzi et al. confirmed the impact of retrograde perfusion on the neurologic outcome (27). This possibility is rather an inherent issue of the MIMVS procedure than associated with the risk factor *previous sternotomy*. Our early postoperative results display an excellent result with respect to the neurological outcome (PS group: 0% vs. non-PS group: 3.1%; *p* = 1.000), although all of our patients received retrograde cannulation, and 46.7% patients of our PS group received ventricular fibrillation. One factor that might contribute to this result is the obligatory CT scan during the assessment of our redo patients who are operated on *via* a full sternotomy in case of enlarged calcification.

The increased duration of the ICU stay in the PS group was not significant but was clearly present (PS group: 2 days, 95% CI, 1.0–8.0 vs. non-PS group: 1.0 days, 95% CI, 1.0–2.0; *p* = 0.072). A closer analysis reveals that two cases primarily contributed to this difference. One patient received a bleeding-induced revision, resulting in an ICU stay of 11 days. Another patient suffered a prolonged duration of ventilation (>48 h) because of pulmonary complications, resulting in an ICU stay of 19 days. Considering both causative aspects separately, no significant difference to the non-PS group can be observed. The rate of bleeding-induced revisions in the PS group (13.3%) is higher than in the cohort of Murzi et al. (6.3%) (9), but it is quite comparable to that reported by Seeburger et al. (12%) (8).

We observed excellent results with respect to functional and clinical outcomes in the follow-up analysis, without any significant difference between the groups. The Kaplan–Meier analysis displayed no significant difference regarding the postoperative mortality related to the at-risk patients. Moreover, our PS group displayed a 30-day mortality of 0%, which is an excellent result and definitively comparable to those of Seeburger et al. (6.6%) (8) and Murzi et al. (4.1%) (9).

When discussing alternative approaches such as TMVR, certain benefits are obvious, e.g., the avoidance of mechanical ventilation eliminates prolonged ventilation as a key risk factor in the postoperative course. However, Taramasso et al. (28) demonstrated in a cohort of patients with functional MV regurgitation that relapse of severe MV regurgitation occurs in up to 20% of patients at 1 year after TMVR. Compared with the results in a parallel group of patients receiving surgical MV repair, this result was somewhat inferior with respect to functional outcome (*p* = 0.001). Such findings mirror our own regarding excellent functional MV competence 1 year after surgery and clearly emphasize the value of current surgical techniques.

## Conclusion

A previous sternotomy remains a risk factor for MIMVS that demands special attention in the early postoperative period. Nevertheless, in a direct propensity-matched comparison to patients without a previous sternotomy, parameters of the surgical procedure, postoperative course, functional outcome, and mortality display no inferiority. The risk factor, i.e., *previous sternotomy*, should not be considered as the sole determinant for or against a particular technique.

### Limitations

Our study is limited by being a retrospective, single-center experience and by its small number of patients in the group with a previous sternotomy (PS group).

## Ethics Statement

This study was approved by the Ethics Committee of the Medical Faculty of the Heinrich Heine University of DUesseldorf with written informed consent from all subjects. All subjects gave written informed consent in accordance with the Declaration of Helsinki.

## Author Contributions

Conceptualization: J-PM. Data curation: J-PM and VD. Formal analysis: J-PM. Investigation: VD, PR, and SS. Methodology: J-PM, VD, PR, and SS. Project administration: J-PM and PA. Resources: PA, AA, HK, and AL. Supervision: PA, UB, and AL. Validation: J-PM and PA. Visualization: J-PM and VD. Writing (original draft preparation): J-PM, PA, and UB. Writing (review and editing): J-PM, PA, UB, and AL.

## Conflict of Interest Statement

The authors declare that the research was conducted in the absence of any commercial or financial relationships that could be construed as a potential conflict of interest.
