# Supplementary figures and images for: Previous Sternotomy as a Risk Factor in Minimally Invasive Mitral Valve Surgery
Source: Front Surg. 2018 Feb 9;5:5. doi: 10.3389/fsurg.2018.00005 (PMC5811546; doi:10.3389/fsurg.2018.00005)

## Slide 1
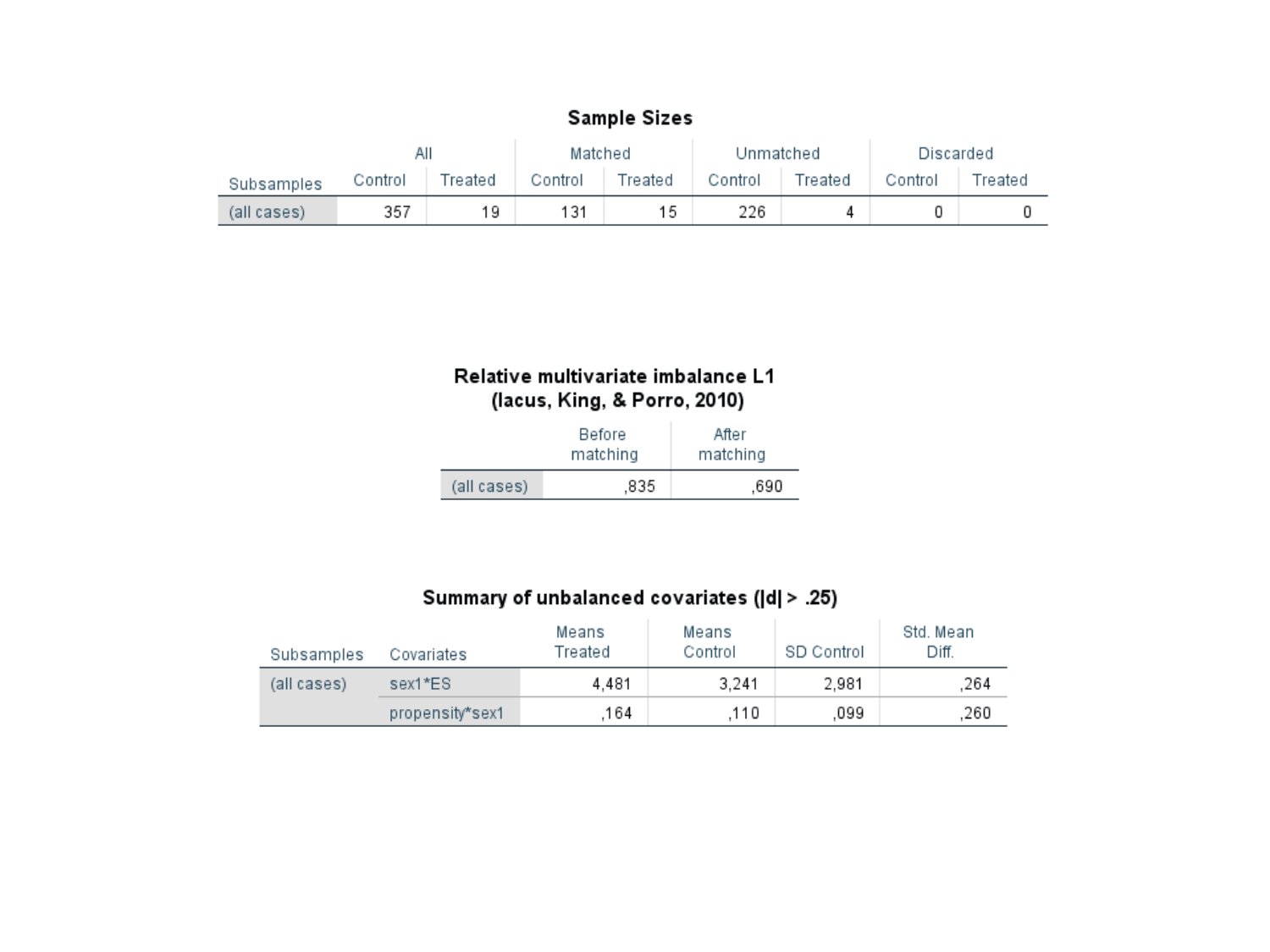

## Slide 2
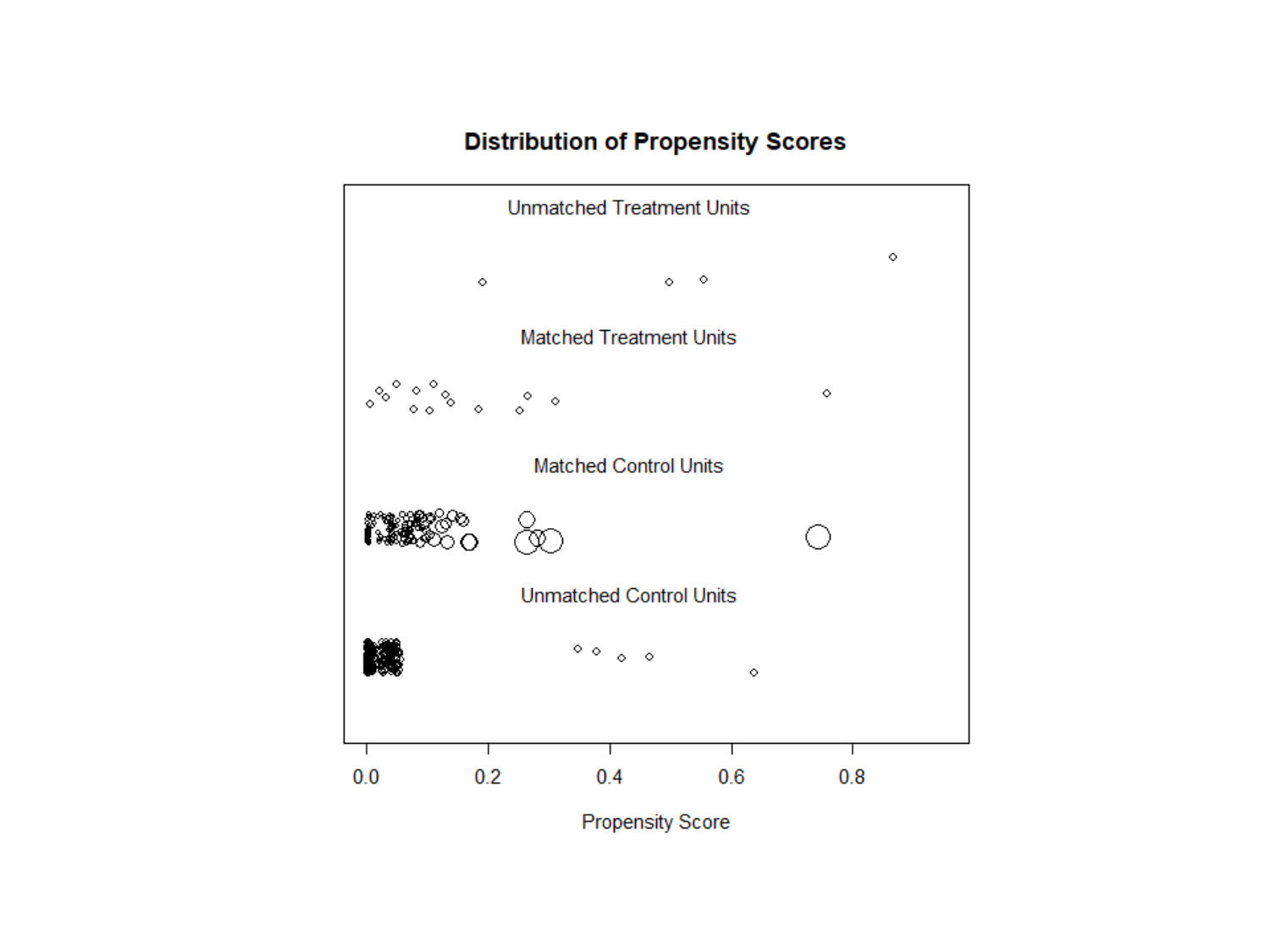

## Slide 3
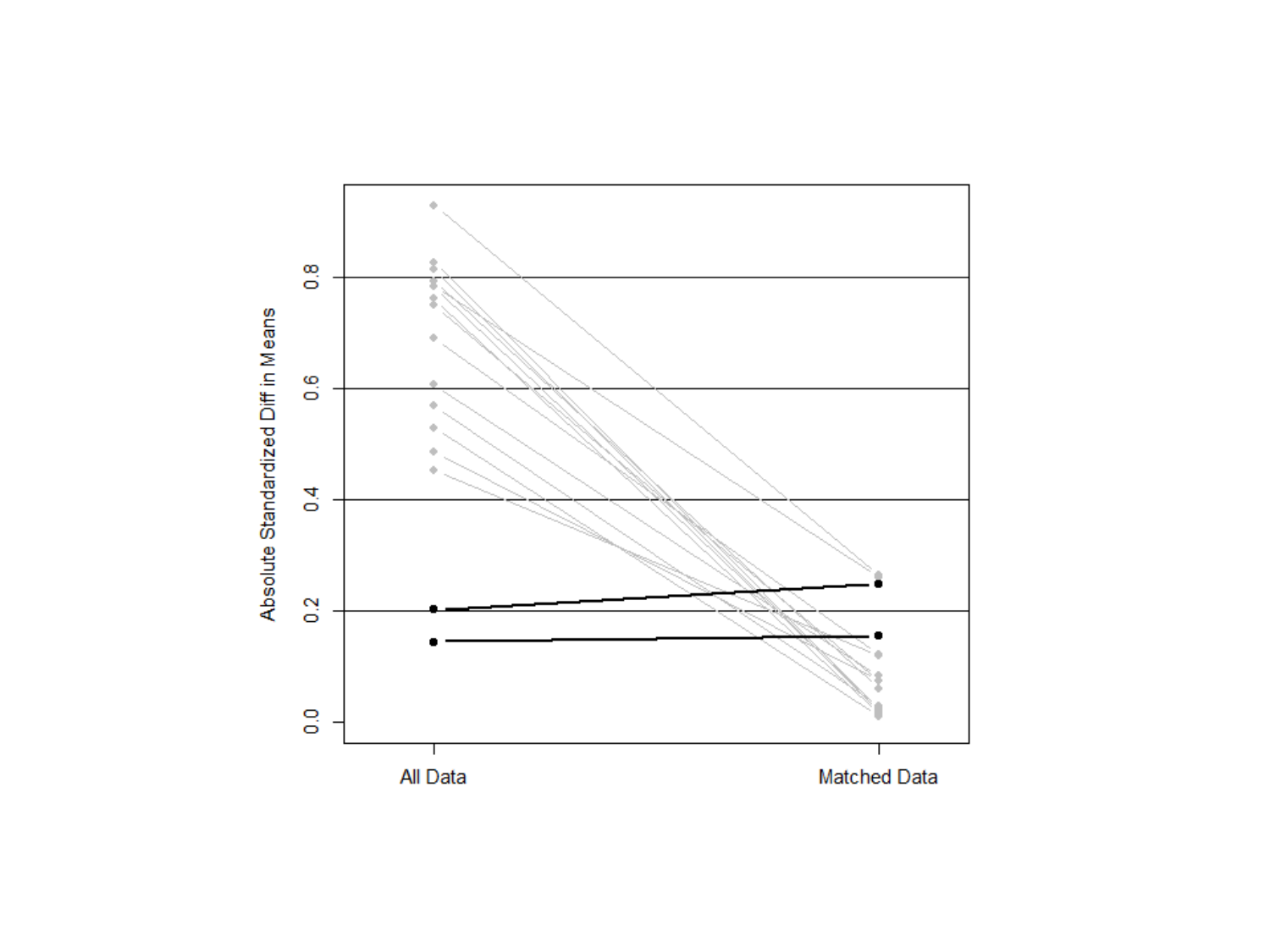

## Slide 4
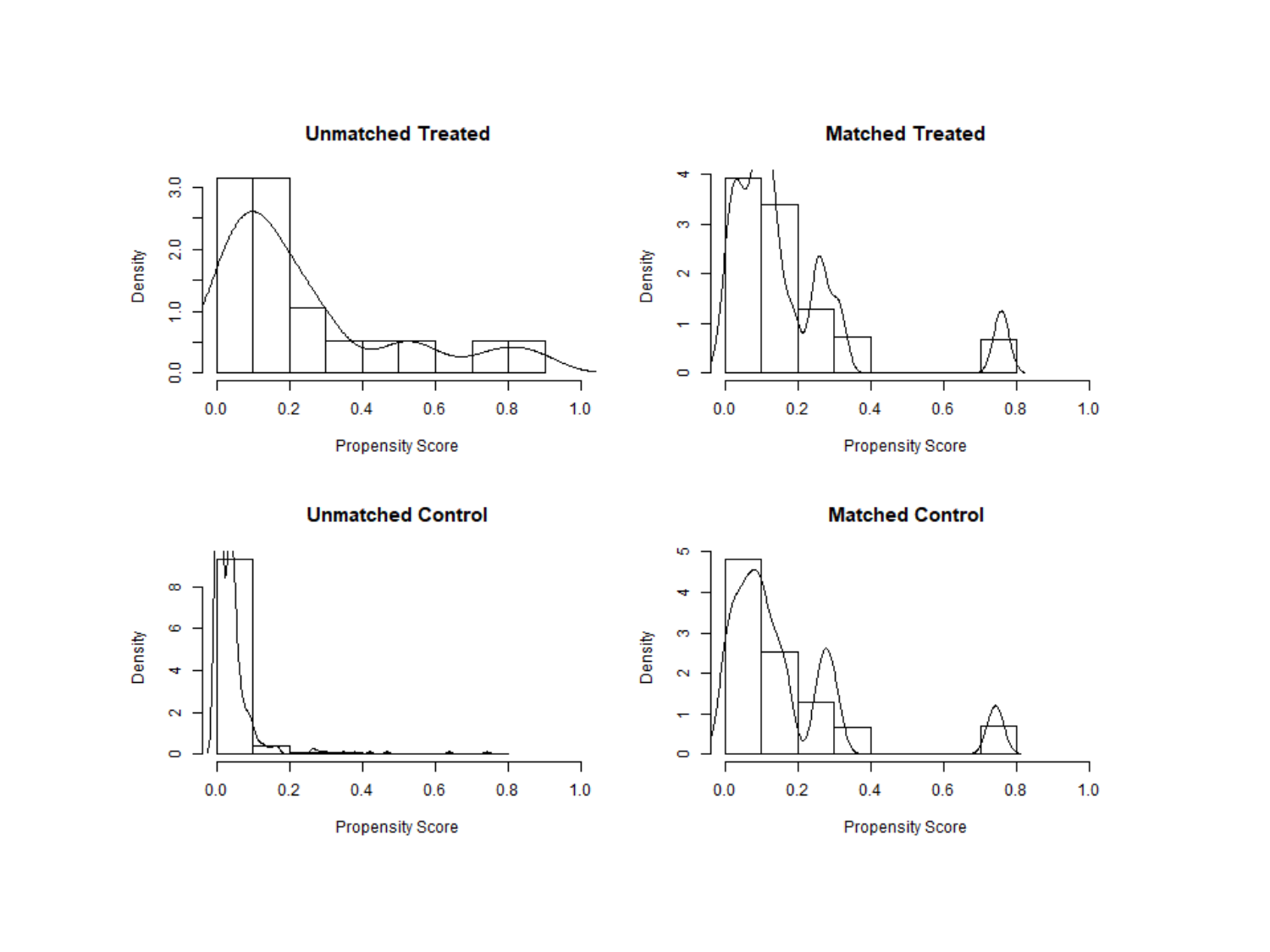

## Slide 5
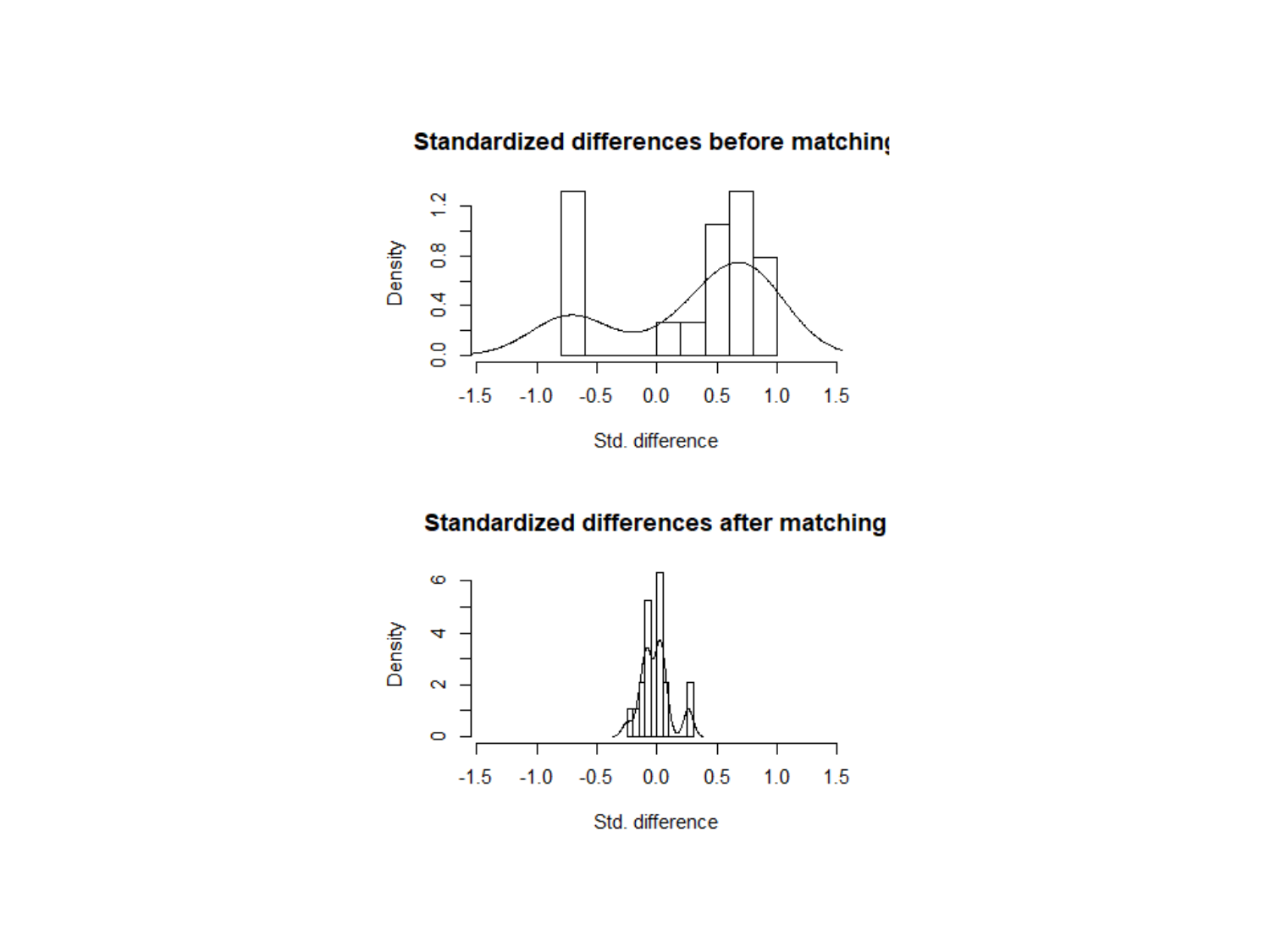

## Slide 6
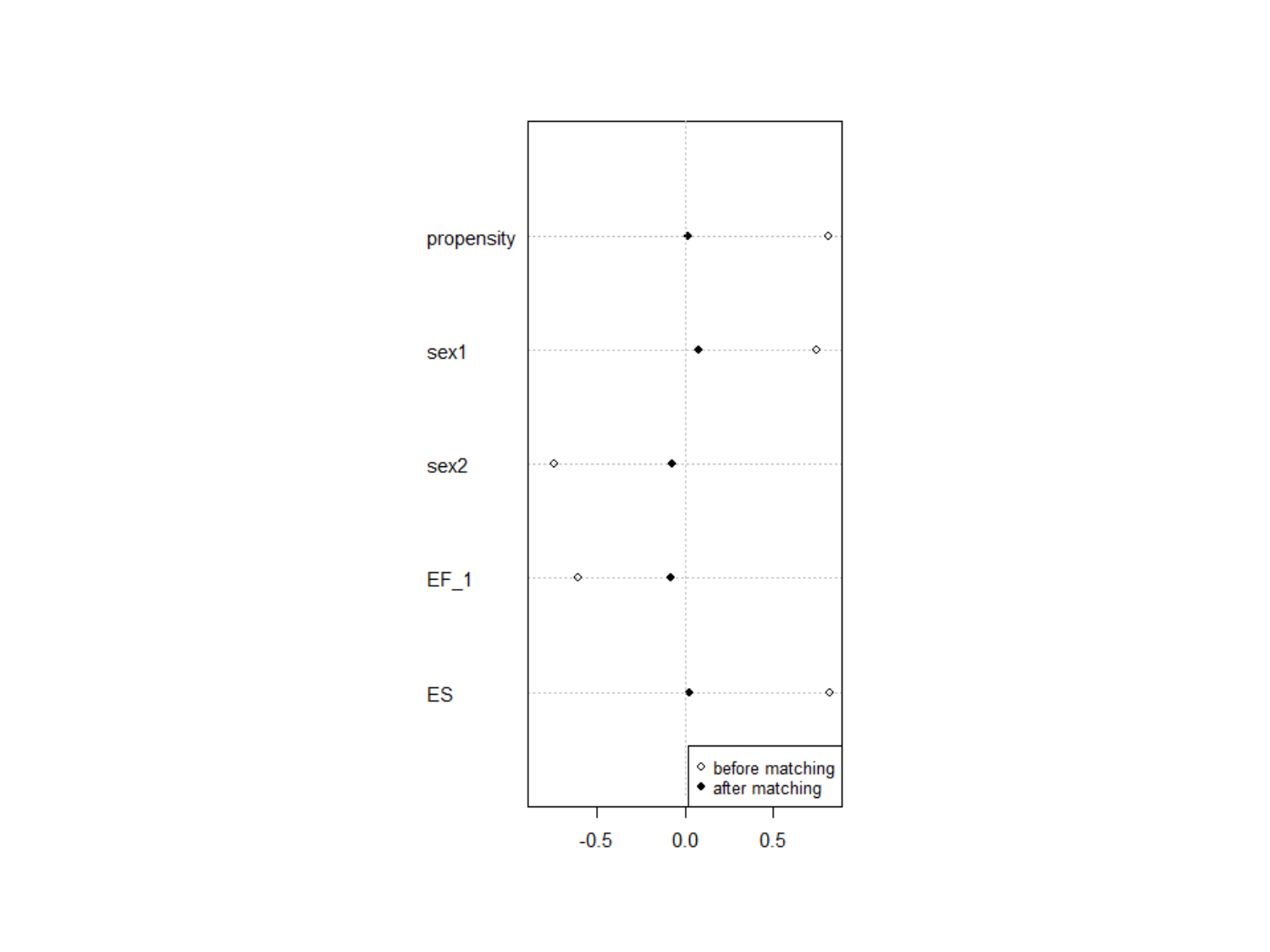

Supplement: Supplementary file 1 [file Presentation_2.pptx]
